# Cockchafer Larvae Smell Host Root Scents in Soil

**DOI:** 10.1371/journal.pone.0045827

**Published:** 2012-10-01

**Authors:** Sonja Weissteiner, Wolf Huetteroth, Martin Kollmann, Bernhard Weißbecker, Roberto Romani, Joachim Schachtner, Stefan Schütz

**Affiliations:** 1 Forest Zoology and Forest Protection, Georg-August-Universität, Göttingen, Germany; 2 Neurobiology/Ethology, Philipps-University, Marburg, Germany; 3 Dipartimento di Scienze Agrarie e Ambientali, University of Perugia, Perugia, Italy; AgroParisTech, France

## Abstract

In many insect species olfaction is a key sensory modality. However, examination of the chemical ecology of insects has focussed up to now on insects living above ground. Evidence for behavioral responses to chemical cues in the soil other than CO_2_ is scarce and the role played by olfaction in the process of finding host roots below ground is not yet understood. The question of whether soil-dwelling beetle larvae can smell their host plant roots has been under debate, but proof is as yet lacking that olfactory perception of volatile compounds released by damaged host plants, as is known for insects living above ground, occurs. Here we show that soil-dwelling larvae of *Melolontha hippocastani* are well equipped for olfactory perception and respond electrophysiologically and behaviorally to volatiles released by damaged host-plant roots. An olfactory apparatus consisting of pore plates at the antennae and about 70 glomeruli as primary olfactory processing units indicates a highly developed olfactory system. Damage induced host plant volatiles released by oak roots such as eucalyptol and anisol are detected by larval antennae down to 5 ppbv in soil air and elicit directed movement of the larvae in natural soil towards the odor source. Our results demonstrate that plant-root volatiles are likely to be perceived by the larval olfactory system and to guide soil-dwelling white grubs through the dark below ground to their host plants. Thus, to find below-ground host plants cockchafer larvae employ mechanisms that are similar to those employed by the adult beetles flying above ground, despite strikingly different physicochemical conditions in the soil.

## Introduction

Soil-dwelling animals face the challenge of how to move towards vital resources, including food, in a dark environment, where every movement in the soil matrix consumes substantial amounts of energy. Even if the female adult can lay eggs close to host trees, limiting the needs for larvae to move in the soil [1], [2], the larvae need to undertake directed movements towards the desired resource in order to keep up with the dynamics of the permanently changing root system of a tree [3]. Contact-mediated perceptions such as the tactile and gustatory senses deliver only close-range information about the highly heterogeneous soil environment. Olfactory or auditory senses can mediate information on resources beyond immediate contact and provide some clue as to the direction of most rewarding movement [1], [2], [4], [5]. Above-ground olfaction is the prime sensory perception guiding especially night-active insects towards vital resources [6]. However, the question arises as to the suitability of this sensory modality for locating resources below ground. Are volatile organic compounds (VOC) which might serve as odors present in soil? Do soil-dwelling insect instars possess sensory structures and brain structures to perceive and to process these VOCs as clues? Can their behavioral patterns, mediated by these perceptions, be linked to meaningful resource location?

Natural soil is a complex multiphase-system consisting of organic and inorganic compounds as solid phase components, adsorbed and free water as liquid phase components, and dissolved and free air as gas phase components. Thus, volatile organic compounds are part of the soil, adsorbed at solid phase particles, dissolved in soil water, and evaporated as a part of the soil air. In contrast to circumstances above ground, VOCs in soil are not transported rapidly over long distances by wind, but are slowly transported by diffusion processes in soil capillaries. During this slow transport, VOCs in soil come into contact with capillary walls increasing the probability of interaction processes like adsorption or chemical degradation. This might result in a strongly reduced range of transport of heavy, reactive VOC molecules in soil [7], [8].

Larvae of the cockchafers of the genus *Melolontha* (Coleoptera: Scarabaeidae) cause conspicuous root damage to a range of horticulturally and silviculturally important plants, especially to young oaks on reforestation sites. While the adult beetles migrate kilometers and defoliate several tree species, in later developmental stages the grubs show a pronounced preference for a variety of tree roots and are able to move as far as several meters through the soil towards their targets [9]. In fact, there are several indications that carbon dioxide is an important clue for the white grub seeking to locate respirating plant roots as a food resource [9]. However, decaying roots also release substantial amounts of carbon dioxide without being a food resource. Moreover, differentiation of host-plant and non-host-plant roots needs additional information. VOCs released specifically by suitable plant roots might provide an additional clue [2]. However, the role of plant-root-borne VOCs in respect to *Melolontha* larvae is not yet clear. The curculionid beetle larva *Hylobius abietis* specifically attacks roots of stressed spruce trees. In choice tests without soil, ethanol and *a*-pinene were observed to elicit chemotactic behavior by *Hylobius abietis*, providing the first hint that VOCs may mediate food resource location behavior in soil [10], [11].

In the present study, VOCs released by intact and damaged oak roots were analyzed. Basing on gaschromatography-mass spectrometry with parallel electroantennographic detection (GC-MS/EAD) [12], morphological and electrophysiological examinations were performed to check whether the soil-dwelling larvae of the forest cockchafer (*M. hippocastani*) possess sensory structures to perceive host-plant root-derived VOCs. Brain morphological studies were performed to identify brain structures responsible for processing these VOCs as odors. And lastly, behavioral choice assays in soil were used to check whether the perception of host-plant root VOCs could mediate significant chemotactic behavior and could be linked to differentiated host-plant root location.

## Materials and Methods

### Insects

Second instar larvae of *M. hippocastani* were collected in late May of 2007 in a forest near Darmstadt (Germany). The larvae were kept in the dark for 4–7 months at 20°C. To prevent cannibalism, larvae were kept individually in small boxes (250 ml) filled with sieved native soil from Darmstadt. At once- to twice-weekly intervals, the humidity of the substrate was checked and the larvae were fed with fresh slices of carrot. Old carrot slices were removed. Only actively feeding insects of the 2^nd^ and 3^rd^ instar were used for the experiments.

### Plants

120 young oak trees (*Quercus petraea* × *Quercus robur* ) from a forest near Göttingen (Germany) were kept from 2004–2007 in a greenhouse in original soil substrate under controlled conditions (photoperiod 16 hours, 10 kLux, 19–25°C, 40–50% relative humidity, individual buckets). During the winter period the oaks were transferred outside, where the pots were embedded in the soil to prevent the roots from frost damage within the soil.

### Sampling of Volatiles

One larva of *M. hippocastani* was placed in each pot in 10 cm depth and 10 cm radial distance to the roots of the oak trees (feeding damage treatment, N = 20), to enable feeding on the roots. After 48 h soil air was sampled in 10 cm depth and 10 cm distance of the roots from 10 oak trees with feeding larva and from 10 oak trees without feeding larva by a passive sampling method (1 h SPME, Carboxen/polydimethylsiloxane, Supelco/Sigma-Aldrich, Taufkirchen, Germany) and an active sampling method (TDS, Gerstel, Mühlheim, Germany) for 1 hour with a flow rate of 0.1 l/min [13].

Prior to measurement of washed roots, rinsing was carried out using tap water and the soil was carefully removed. Roots with visible feeding traces were defined as roots with feeding damage. 40 trees were left without larvae as undamaged roots. A subset of the undamaged, washed roots was mechanically damaged by cutting the roots using a pair of scissors (mechanical damage, 1% damaged surface within 5 min., measurement 30 min. after damage, N = 20). Samples of root volatiles were obtained using dynamic headspace sampling from humid roots of undamaged trees at daylight conditions. Roots were enclosed into bags of PET-foil (Toppits®, Cofresco Frischhalteprodukte, Minden, Germany). The air was circulated by miniature pumps (Fürgut, Tannheim, Germany) through adsorbent traps loaded with 1.5 mg charcoal (CLSA-Filter, Daumazan sur Arize, France) for 3 hours with a flow rate of 1 l/min in order to get samples suitable for GC-MS/EAD analysis. Volatiles were eluted from the charcoal with 75 µl of a 2+1 mixture of methylene chloride and methanol (both solvents Suprasolv-quality, Merck/VWR, Darmstadt, Germany). Thus, one sample could be measured repeatedly on different larval antennae with each injection into the GC amounting only to 1 µl eluate. In order to get comparable results to measurements in soil, passive sampling of volatile organic compounds was performed by exposing SPME-fiber (Carboxen/polydimethylsiloxane, Supelco/Sigma-Aldrich, Taufkirchen, Germany) for 1 hour to sample air in the PET bags without any forced air movement. Additionally, the air was circulated through Tenax® adsorbent traps (TDS, Gerstel, Mülheim, Germany) for 1 hour with a flow rate of 0.1 l/min. The traps were connected by flexible tubes of Teflon (5 mm ID) to the PET bags.

Soil air from the choice test arena was sampled at the starting point of the larva for 1 h by a passive sampling method (SPME, Carboxen/polydimethylsiloxane, Supelco/Sigma-Aldrich, Taufkirchen, Germany, for 1 h) and an active sampling method (TDS, Gerstel, Mülheim, Germany, for 1 hour with a flow rate of 0.1 l/min) [13].

The samples were stored at −76°C in an ultra low temperature freezer until analysis.

### Gas Chromatography-mass Spectrometry and Analytical Conditions

The root volatiles were analyzed using a gas chromatograph coupled to a quadrupole mass spectrometer (6890N and 5973, Agilent, Santa Clara, USA, technical details: see [12]). For compound identification a nonpolar column (HP-5MS, length 30 m, ID 0.25 mm, and film thickness 0.25 µm, Agilent) and a polar column (INNOWAX, length 30 m, ID 0.25 mm, and film thickness 0.25 µm, Agilent) were used.

One microliter of the eluate was injected into the GC-injector in the pulsed splitless mode (pulse pressure 150 kPa until 1.5 min) at a temperature of 250°C. The samples on the TDS-traps were thermodesorbed in a TDS 2 system (Gerstel, Mülheim, Germany) by heating at 280 C° for 3 min with a helium flow of 40 ml/min. Volatiles were cryo-focussed on a cold injection system CIS 4 (Gerstel) at −75°C. The samples on SPME-fibers were thermodesorbed in the split/splitless injector (splitless mode) at 250°C for 1.5 min. With the nonpolar column, the GC was operating in the following temperature program: 40°C, hold for 2.5 min, ramp 6.2°C/min to 250°C, hold for 10 min. With the polar column the following parameters were used: 50°C, hold for 2.5 min, ramp 7.5°C/min to 250°C, hold for 5 min. Helium (purity 99.999%) was used as carrier gas after passing through a combined adsorbent trap for removal of traces of water, oxygen and hydrocarbons (“Big Universal Trap”, Agilent). The carrier gas flow was set to 1 ml/min resulting in a gas vector of 24 cm/s. The GC-MS interface was operated at a temperature of 280°C.

The mass spectrometer used electron ionization (EI) at 70 eV and was operated in the scan mode with a mass range from 35–300 atomic mass units at a scan speed of 2.78 scans per second.

Selected CLSA samples were examined by a gas-chromatographic set-up with parallel mass spectrometric and electroantennographic detection according to [12].

The preliminary peak identification of the odor compounds was carried out by using the Mass Spectral Search library (version 2005) of the National Institute of Standards and Technology (NIST, Gaithersburg, USA) and the MassFinder 3.0 software together with the library “Terpenoids and Related Constituents of Essential Oils” (Hochmuth, König, Joulain, Hamburg, Germany). The identification of the compounds was confirmed by comparing mass spectra and retention times to those of authentic standards. Selected compounds were quantified based on a standard curve obtained by injecting different concentrations of pure standards [13].

After checking heteroscedascity, root volatile data were compared either by Student’s t (t-test) and one-way ANOVA or by Mann-Whitney U-tests or Kruskal-Wallis ANOVA on ranks (H-tests). Pairwise comparisons were obtained by Tukey’s HSD tests and Dunn’s tests, respectively.

### Electroantennogram (EAG) Dilution Series

**Figure 1:**
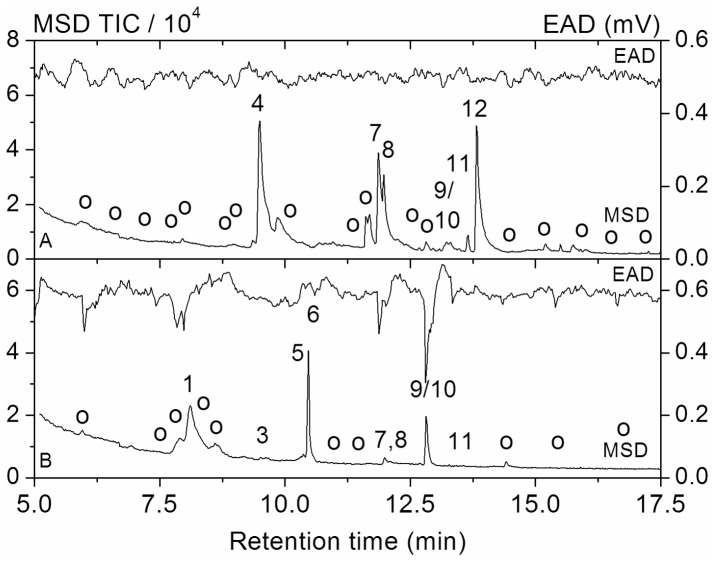
GC-MS/EAD chromatograms of CLSA-samples from oak roots. A) undamaged oak roots The upper trace shows the electroantennographic response of a larval antenna (EAD), the lower trace shows the total ion current of the mass spectrometer (MSD). Compounds detected consistently (numbers as in Table 1): 4∶6-methyl-5-hepten-2-one, 7: furanoid trans-linalooloxide, 8: nonanal, 9/10: (R/S) camphor, 11: borneol, 12: decanal. Not consistently detected compounds are labeled by o. B) feeding damaged oak roots The upper trace shows the electroantennographic response of a larval antenna (EAD), the lower trace shows the total ion current of the mass spectrometer (MSD). Compounds detected consistently (numbers as in Table 1): 1: anisol, 3: octan-3-one, 5∶2-ethyl-hexan-1-ol, 6: eucalyptol, 7: furanoid trans-linalooloxide, 8: nonanal, 9/10: (R/S) camphor, 11: borneol. Not consistently detected compounds are labeled by o.

The results from measurements with a gas chromatograph coupled to an electroantenno-graphic detector and a mass spectrometer (GC-MS/EAD) were the basis for selecting the compounds for further electrophysiological experiments (Table 1, Figure 1). Thus, dilution series from 10^−7^ to 10^−2^ of anisol, eucalyptol, octan-3-one, (1R)-camphor and the furanoid form of trans-linalooloxide were prepared in silicon oil (Carl Roth GmbH + Co. KG, Karlsruhe, Germany). Volatile dilutions (∼30 µl) were applied to pieces of aluminum foil (9 cm^2^), folded and put in 10 ml glass syringes (Poulten & Graf GmbH, Wertheim, Germany). Humidified air from the EAG-system (23°C, 80% RH) was used to fill the syringes. Inside the air volume of the syringes, the odorant accumulates to a concentration proportional to the concentration of the compound in the solution and its vapor pressure according to Henry’s law. By puffing 5 ml of air over the antenna a reproducible stimulus was provided for at least 40 repeated puffs [14]. Resulting concentrations of stimulus compounds in stimulus air are provided in Table 1. The dilution series were measured by manual injection of these odor standards onto the antenna of *M. hippocastani* contacted to the EAG set-up. Responses of at least three antennae from different individuals were recorded for each tested compound. The dissected antenna was placed in an antenna holder (Prof. Koch, Kaiserslautern, Germany) of acrylic glass [15]. The ends of the antennae were immersed in an electrolyte solution [16]. Antennal responses were electronically amplified by a factor of 100. Response amplitudes to stimulus compounds were subtracted from the response amplitudes to the silicon oil control. Additionally, antennal responses to an anisol dilution of 10^−3^ in silicone oil were measured as positive control to check for reproducibility of the antennal responses. Because absolute values of EAG-responses could vary strongly between different antennae, the mean values of the antennal responses to each compound and concentration were calculated in order to show the typical concentration dependency of the antennal response. Moreover, the lowest dilutions eliciting responses significantly different to baseline noise (according IUPAC-definition) from at least 50% of all responding antennae was calculated as detection limit.

**Table 1:** Concentration mean values (scatter) of root VOCs consistently released by oak roots.

Compound	Nr	Source, Purity^1^ CASNr	Conc.a,b in soil 10 cm from root^2^	Concentrationa,cwashed roots^2^Undam. Mech.dam. Feed.dam.Different letter: Significant difference				Conc.a,d antennal detection limit	Conc.a,b in soil,start pointchoice-test	Vapor pressure [mbar]
anisol	1	Merck, 95% 100-66-3	150 ppbv (20–340)	n.d. A	n.d. A	500 ppbv (90–730) B***	5 ppbv		200 ppbv (140–260)	5.03^a^
(R)-1-octen-3-ol	2	Merck, 98% 3391-86-4	n.d.	n.d. A	n.d. A	12 ppbv° (0–43) B*	1.5 ppbv			1.45^a^
Octan-3-one	3	VWR, 96% 106-68-3	10 ppbv (8–17)	n.d. A	n.d. A	15 ppbv (0–34) B*	3 ppbv		20 ppbv (10–35)	3.15^a^
6-methyl-5-hepten-2-one	4	Fluka, 96% 110-93-0	32 ppbv (25–47)	20 ppbv (0–43) A	37 ppbv (5–73) A	15 ppbv (0–75) A	500 ppbv			
2-ethyl- hexan-1-ol	5	Merck, 94% (97%)104-76-7	n.d.	n.d. A	28 ppbv (5–43) B*	10 ppbv (3–25) B*	320 ppbv			0.32^a^
eucalyptol	6	Aldrich, 99% 470-82-6	10 ppbv (2–18)	n.d. A	n.d. A	450 ppbv (70–750) B***	2.6 ppbv		5 ppbv (1–9)	2.60^a^
Linalool-oxide (furanoid)	7	Fluka, 97% 60047-17-8	n.d.	23 ppbv° (0–54) A	27 ppbv° (0–67) A	36 ppbv° (0–110) A	3 ppbv°		10 ppbv° (7–15)	0.027^b^
nonanal	8	Merck, 95% (97%)124-19-6	21 ppbv (10–36)	107 ppbv (10–230)	75 ppbv (25–176)	15 ppbv (0–45)	87 ppbv			0.87^a^
(1R)-camphor	9	Aldrich, 98% 464-49-3	25 ppbv° (15–47)	6 ppbv° (0–14) A	20 ppbv° (5–43) A	120 ppbv° (0–260) A	3 ppbv		30 ppbv° (23–38)	0.30^b^
(1S)- camphor	10	Merck, 95% 464-48-2	25 ppbv° (15–47)	6 ppbv° (0–14) A	20 ppbv° (5–43) A	120 ppbv° (0–260) A	3 ppbv		30 ppbv° (23–38)	0.30^b^
borneol	11	Aldrich, 99% 464-45-9	n.d.	20 ppbv° (0–43) A	36 ppbv° (10–72) A	50 ppbv° (0–76) A	5 ppbv°			0.053^b^
decanal	12	Acros, 95% 112-31-2	n.d.	56 ppbv (13–134) A	43 ppbv (8–78) A	n.d. B**	20 ppbv			0.21^a^
geranyl acetone	13	Aldrich, 97% 3796-70-1	n.d.	10 ppbv (5–27) A	45 ppbv (0–97) A	16 ppbv (0–80) A	2 ppbv			0.021^b^

As measured in 10 cm distance to feeding-damaged oak roots in original soil.

As measured to be released by washed oak roots (undamaged, mechanically damaged, feeding-damaged).

As lowest concentration stimulating the white grub antenna.

As measured in the center of the choice-test arena within the first hour of experiment.

1 Purity checked by gas chromatography-mass spectrometry, deviations from label given in brackets.

2 Compounds released by *Quercus* spec. roots under special circumstances.

a passive sampling by SPME (n = 3).

b active sampling by TDS (n = 5).

c active sampling by CLSA (n = 9).

d calculated from vapour pressure based on Yaws CL (2007) The Yaws Handbook of Vapor Pressure. Houston: Gulf Publishing Company and based on SciFinder® database https://scifinder.cas.org.

°Racemic Mixture;

n.d. not detected

Different letters indicate significant differences,

* :P<0.05,

** :P<0.01,

*** :P<0.001.

### Behavioral Tests

**Figure 2:**
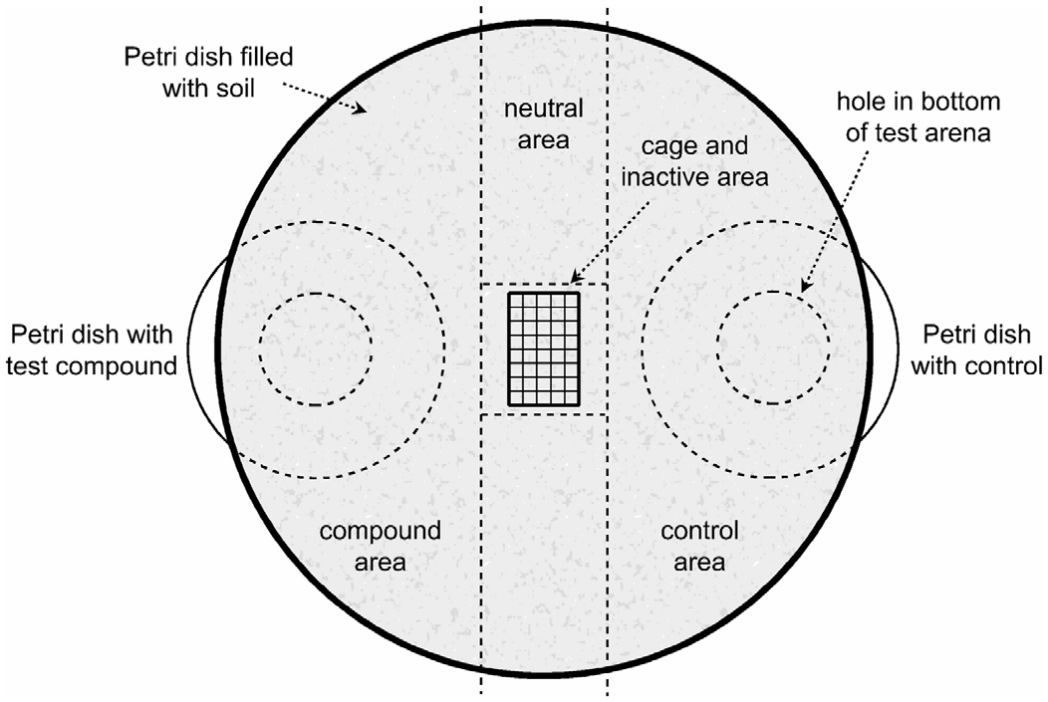
Design of an experimental unit of the dual choice bioassay. The set-up consists of one large Petri dish (ID 14 cm) with two holes (diameter 24 mm each) in the lid, two small Petri dishes (ID 5 to 6 cm) and a cage made of steel wire (2.5 cm×1.5 cm). Larvae chose between the test compound and the control, or stayed in the neutral area (central bar of 3×14 cm, orthogonal to the connection line of the holes). First choice position in relation to the stimulus compound, the control or the neutral area was recorded.

A dual choice bioassay was used for the behavioral part of the experiment. The results from measurements of washed root samples with a gas chromatograph coupled to an electroantennograph and a mass spectrometer (GC-MS/EAD) provided the basis for selecting the compounds for further behavioral experiments. Additionally, concentrations of volatiles measured in soil were compared with the detection limit of the larval antennae to select promising candidate volatiles (Table 1, figure 1). Each experimental set-up consisted of a Petri dish (14 cm ID, 2 cm deep) with the corresponding lid, two smaller Petri dishes without lid (5 to 6 cm ID), and a small cage made of steel gauze (2.5 cm×2.5 cm×1.5 cm). The lid of the Petri dish had two holes at opposite locations (figure 2). For each behavioral experiment a single larva was placed in the centre of the 14 cm Petri dish inside the steel cage and surrounded by sieved, native soil. After at least 15 hours of adaptation, the cage was removed and the Petri dish was turned upside down. The smaller Petri dishes were placed below the holes, with the diluted test compound in one dish and pure silicon oil as the control in the other dish (∼30 µl each). The concentration of each compound within the arena at the starting point of the larvae was measured by passive volatile sampling (SPME). The dilutions were chosen to match the concentrations measured in soil 10 cm from an oak root. As a reference experiment the same set-up was used with the small Petri dishes containing 1 g freshly feeding-damaged oak roots (one L3 larvae fed for 24 h and was then removed) in original soil matrix, and 1 g undamaged roots in original soil matrix each as parts of undamaged trees. The Petri dishes were distributed and oriented randomly to avoid position effects. The experiments were performed immediately after turning the Petri dish in a dark room at 20°C.

In one experimental cycle the choice of ca. 15 single larvae was recorded in parallel. The larvae could opt to move towards the test compound or the control, stay in the central part (a central segment of 3×3 cm), or move within the central band (a central bar of 3×14 cm, orthogonal to the connection line of the holes). The position of the larvae was recorded at 10 to 15 minute intervals. First choice position in relation to the stimulus compound, the control or the neutral area was recorded. The entire experiment was terminated after one hour and the behavior of the larvae towards the odor stimulus was rated in “repellent”, “no decision”, “inactive”, and “attractant”.

The attraction index Attr. I. is the number of larvae in the area of the test compound (first choice) divided by the number of actively deciding larvae. Moreover, the percentage of larvae orienting towards one odor source was calculated. The significance of the results was statistically evaluated by chi^2^ tests.

### Morphological Examination of the Larval Antenna of *M. hippocastani*

#### Scanning electron microscopy (SEM)

Ten *M. hippocastani* 3^rd^ instar larvae were used for the observations. Insects were anesthetized using CO_2_ and kept at −18°C until death. Then, individuals were dissected by removing the antennae from the head capsule. Specimens were dehydrated in a series of graded ethanol (Panreac®, Barcelona, Spain), from 50% to 99% (10 minutes each). After dehydration, the specimens were treated with HMDS (Hexamethyldisilazane, Sigma-Aldrich®, St. Louis, USA) and gold-sputtered using a Balzers Union SCD 040 unit (Balzers®, Vaduz, Luxembourg). On each aluminum stub 5 specimens were mounted, taking care to place them with different orientations in order to obtain a clear view of the ventral, dorsal and both lateral sides. The observations were carried out using a scanning electron microscope Philips XL 30 (FEI® Company, Eindhoven, The Netherlands).

#### Transmission electron microscopy (TEM)

Ten *M. hippocastani* 3^rd^ instar larvae were anesthetized with CO_2_ and immediately immersed in a solution of glutaraldehyde and paraformaldehyde (Electron Microscopy Sciences®, Hatfield, USA) 2.5% in cacodylate (TAAB®, Berks, UK) buffer +5% sucrose (TAAB®, Berks, UK), pH 7.2–7.3. In order to achieve optimal fixation, the last antennomere was detached from the rest of the antenna to help fixative penetration, and left at 4°C for 2 hours. After rinsing overnight in cacodylate buffer, the specimens were post fixed in 1% osmium tetroxide (Electron Microscopy Sciences®, Hatfield, USA) for 1 h and rinsed in the same buffer. Dehydration in a graded ethanol series was followed by embedding in Epon-Araldite (Fluka® Analytical - Sigma Aldrich®, Steinheim, Germany) with propylene oxide (TAAB®, Berks, UK) as bridging solvent. Semi-thin and thin sections were taken with a diamond knife (Drukker®, Cuijk, The Netherlands) on a LKB® Nova ultramicrotome (LKB®, Bromma, Sweden), and mounted on formvar (Serva®, Heidelberg, Germany) coated 50 mesh grids (SPI®, West Chester, USA). Finally, the sections were investigated with a Philips EM 208 (FEI® Company, Eindhoven, The Netherlands), after staining with uranyl acetate (Fluka® Analytical - Sigma Aldrich®, Steinheim, Germany) (20 min, room temperature) and lead citrate (Fluka® Analytical - Sigma Aldrich®, Steinheim, Germany) (5 min, room temperature). Digital pictures (1376×1032 pixels, 8 bit, uncompressed grey scale Tiff files) were obtained using a high resolution digital camera MegaView G2 (SIS® - Olympus®, Tokyo, Japan) connected to the TEM.

#### Immunocytochemistry and antennal backfills

To selectively label neuropil structures in 3^rd^ larval instars of *M. hippocastani* including olfactory glomeruli (see e.g. [17]) we used a monoclonal antiserum from mouse against the ubiquitous synaptic vesicle protein synapsin I (SYNORF1, kindly provided by Dr. E. Buchner, University of Würzburg, Germany; [18]). For whole-mount staining we adapted the staining protocol described in [19]. Whole brains were dissected under cold phosphate buffered saline (PBS 0.01 M, pH 7.4) and subsequently fixed at 4°C overnight in a solution composed of one part formaldehyde (37%, Roth, Karlsruhe, Germany), one part methanol, and eight parts PBS 0.01 M. The brains were then rinsed in 0.01 M PBS for 1 hour at room temperature followed by preincubation overnight at 4°C in 5% normal goat serum (NGS; Jackson ImmunoResearch, Westgrove, PA) in 0.01 M PBS containing 0.3% Triton X-100 (PBST). The synapsin I antibody was diluted 1∶100 in PBST containing 1% NGS: in this solution the brains were incubated for 5 to 6 days at 4°C. Subsequently the brains were rinsed six times in 2 hours with PBST before being incubated with the secondary goat anti mouse antibody conjugated to Cy2 (1∶300, Jackson ImmunoResearch) in PBST and 1% NGS for 4 days at 4°C. After this time the brains were rinsed again with PBST six times in 2 hours. Thereafter the brains were dehydrated in an ascending alcohol series (50% to 100%, 10 minutes each) and then cleared in methyl salicylate (Merck, Gernsheim, Germany) for about 40 minutes. Finally, the brains were mounted in Permount (Fisher Scientific, Pittsburgh, PA) between two coverslips using three spacers (Zweckform, Oberlaindern, Germany) to prevent compression of brains.

Antennal backfills were performed according to the method described in [20]. Crystals of biotinylated dextran (lysine-fixable, molecular mass 3000 Da; Molecular Probes, Eugene, OR, USA) were placed on the cut ends of one antenna of immobilized L3 larva. The antennal stump was sealed with vaseline. The animal was kept in a humid chamber overnight at 4°C to allow the dextran to diffuse through the antennal nerve into its target area in the brain. The next day animals were dissected and the brains were processed for immunocytochemistry as described above. Dextran was visualized using Cy3-coupled streptavidin (1∶300, Jackson Immuno Research), which was applied for 1 h at room temperature.

Fluorescence was analyzed using a confocal laserscan microscope (Leica TCS sp2). The wholemount preparations were scanned at 512×512 pixel resolution by using a 20× oil immersion objective (HC PL APO 20×/0.70 lmm Corr CS; Leica, Bensheim, Germany). All brains were scanned with a voxel size of 0.73×0.73×1 µm, a speed of 200 Hz, a pinhole of 1 Airy unit and a line average of 2 to 4.

## Results

### Volatile Compounds of *Quercus*- Roots

A total of 60 volatile organic compounds were identified by GC-MS of undamaged roots of *Quercus*, mechanically damaged roots, and roots damaged by larval feeding of *M. hippocastani*. The pattern of these volatiles showed a high variation. However, 13 of the identified compounds were consistently detected in the different treatments (Table 1) and contributed 30–90% of the total peak area. Seven different volatile organic compounds were detected in undamaged oak roots, 9 compounds in mechanically damaged roots, and 12 in roots damaged by larval feeding.

The alcohols 1-octen-3-ol and anisol, the ketone octan-3-one and the monoterpenoid eucalyptol were found only in the samples damaged by larval feeding, whereas decanal was found in undamaged and mechanically damaged roots. 2-ethyl-1-hexanol appeared in mechanically damaged oak-roots and in those which were damaged by feeding of the larvae. Furanoid trans-linalooloxide, nonanal, camphor and borneol could be found in all treatements.

The concentrations of anisol and eucalyptol measured in the washed root experiment on feeding-damaged roots translate into emission rates of 24.1+/−7.3 ng h^−1^ g(FW)^−1^ and 31.0+/−7.8 ng h^−1^ g(FW)^−1^.

### Electrophysiological Response of *M. hippocastani* to VOCs Released from Oak Roots Damaged by Larval Feeding

**Figure 3:**
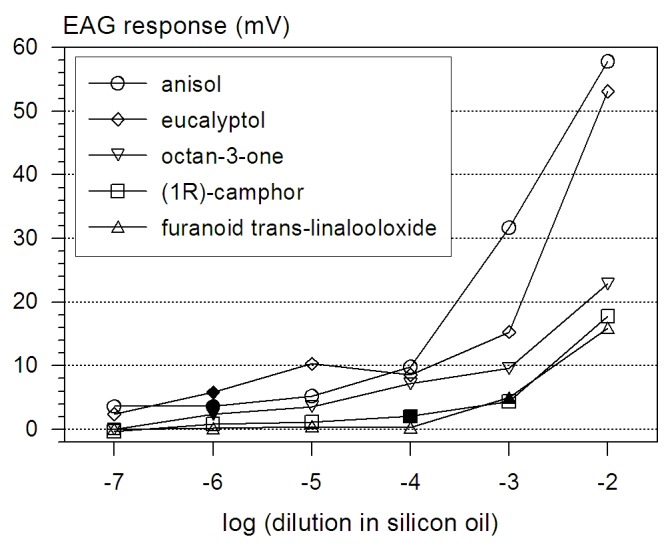
Dose-response curves of *M. hippocastani* to different odors. The selected test odors are released by oak-roots damaged by feeding larvae. Dose- response curves to anisol (N = 8), eucalyptol (N = 3), octan-3-one (N = 6), (1R)-camphor (N = 8), and furanoid trans-linalooloxide (N = 3); mean values of antennal responses (amplification factor 100). Lowest dilutions eliciting responses significantly different to baseline noise from at least 50% of all antennae are marked as full symbols.

The five compounds physiologically active in GC-MS/EAD-experiments at natural concentration levels, anisol, eucalyptol, octan-3-one, (1R)-camphor and furanoid trans-linalooloxide were investigated further for quantitative electrophysiological response (Table 1, figure 3). The first three compounds were released particularly from oak-roots damaged by larval feeding of *M. hippocastani* larvae; the last two were released by all oak roots examined. The highest response (amplification factor 100) to a puff in the dilution 10^−2^ g/g was observed for the alcohol anisol and the monoterpenoid eucalyptol; for anisol the response was 58 mV (±16 mV) on average, for eucalyptol approximately 34 mV (±13 mV). The mean response to the two ketones octan-3-one and camphor was 23 mV (±7 mV) and 17 mV (±4 mV), respectively, and the mean response to the furanoid form of trans-linalooloxide was 16 mV (±2 mV). For camphor we tested the two enantiomers and we could not observe significantly different responses from the antennae. The detection limit varied among the tested compounds (figure 3, Table 1).

### Functional Anatomy of the Antennal Olfactory Sensilla of *M. hippocastani* Larva

**Figure 4:**
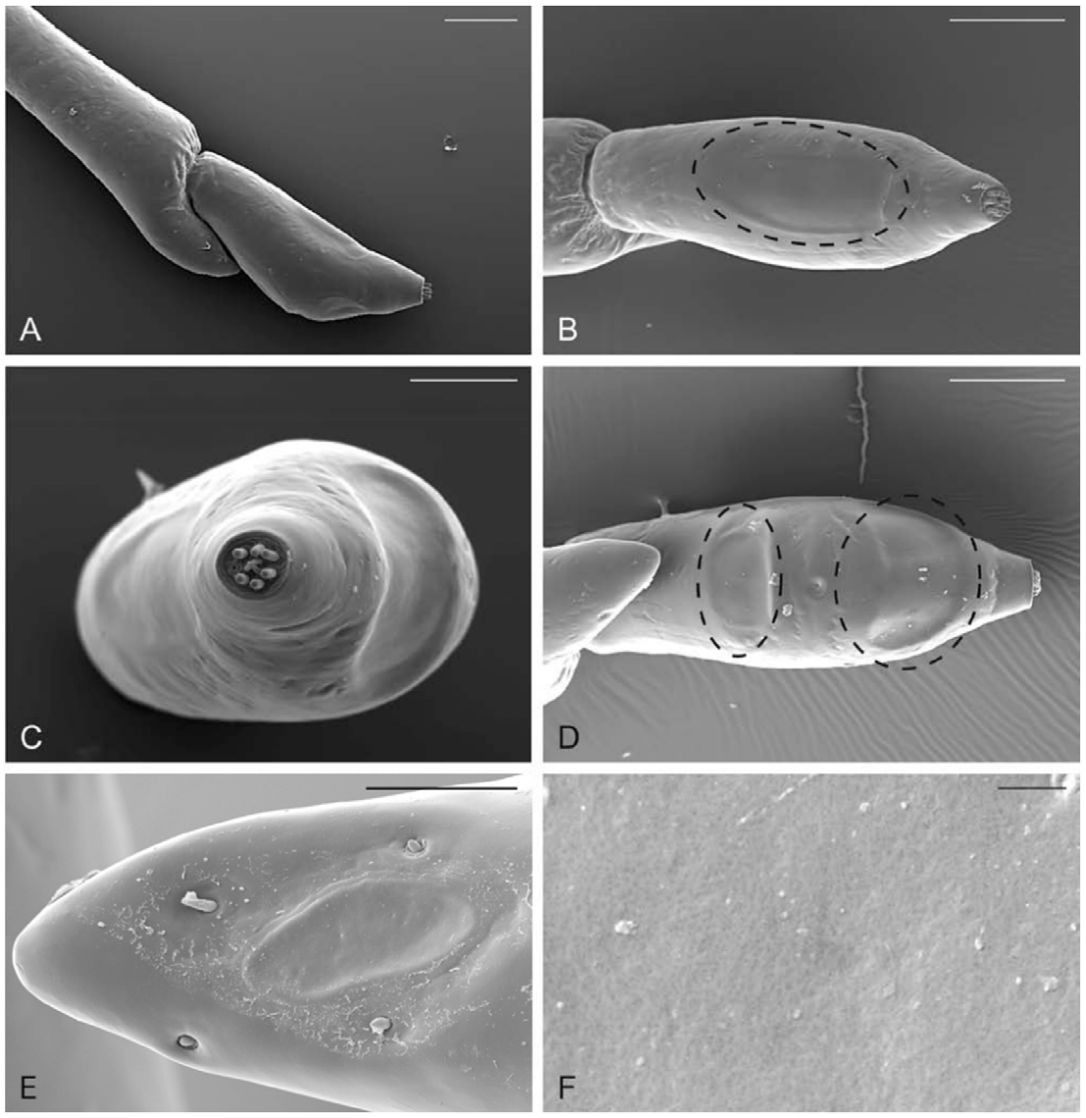
SEM pictures of *M. hippocastani* apical and subapical antennomere. A) Lateral view of the apical and sub-apical antennomere. B–D) Dorsal, apical, and ventral view of the apical antennomere, respectively. In B and D the multiporous olfactory sensilla (MOS) can easily be observed. In C the apical part of the antennomere is shown, with the dorsal (left) and ventral (right) MOS. E) MOS on the inner surface of the lateral protrusion of the subapical segment. F) Close up view of the MOS surface, pierced by numerous tiny cuticular pores. Bar scale: A, B, D: 200 µm; C:100 µm; E: 50 µm; F: 2 µm.

The antennae of *M. hippocastani* larvae were found to consist of 4 antennomeres. The apical antennomere was shorter than the sub-apical and had a typical triangular shape (when observed from one of the external sides) (figure 4 A). Apically, the antennomere displayed a specialized, truncated area housing 10 pegs of various shapes (figure 4 C). These sensilla showed ultrastructural features unrelated to olfactory sensilla, such as a poreless cuticle and the absence of a single apical pore, strongly indicating that they were not involved in olfaction (data not shown). External observations of the long (dorsal) and short (ventral) side revealed the presence of three smooth, slightly depressed areas (figure 4 B–D). The dorsal area was more rectangular in shape (figure 4 B), while the two ventral areas were sub-elliptical (figure 4 D). The average total surface area occupied by the three sections was about 90,000 µm^2^. One slightly oval pore plate of 30 µm in width and 70 µm in length was located on the inner surface of the lateral protrusion of the subapical segment (figure 4 E).

**Figure 5:**
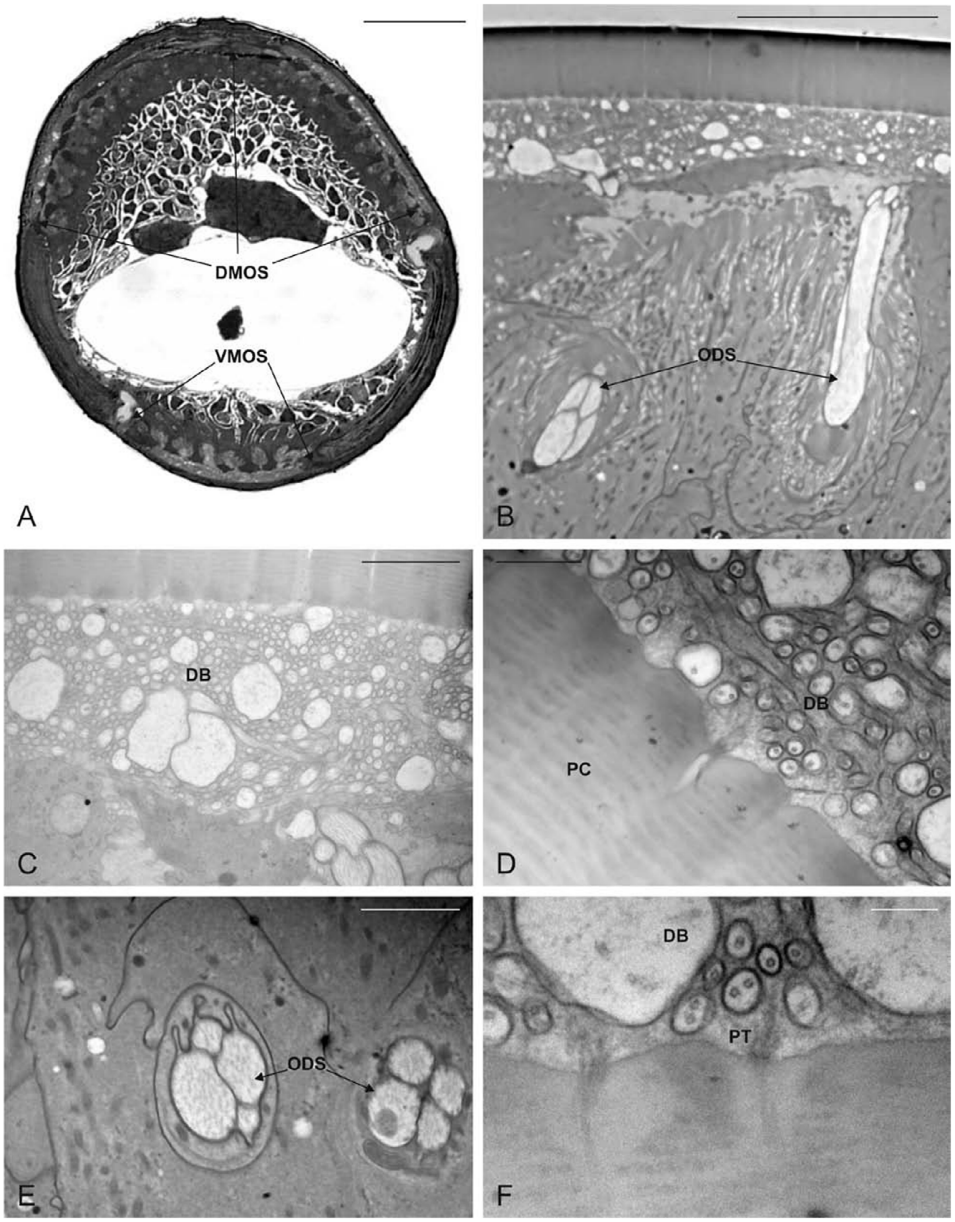
*M. hippocastani* apical antennomere. A) Light microscopy cross section showing the dorsal (DMOS) and ventral MOS (VMOS). B) TEM cross section at the level of the dorsal MOS, showing two bundles of outer dendritic segments (ODS). C, D, F) Details of the dendritic branches (DB) filling the space below the porous cuticle (PC), pore tubules (PT) can also be observed. E) Two bundles of four dendrites taken at the level of the ODS. Bar scale: A: 50 µm; B: 10 µm; C, E: 2 µm; D: 500 nm; F: 200 nm.

SEM high magnification images showed the presence of numerous scattered tiny pores evenly distributed on the whole surface (figure 4 F). Light and TEM serial cross section revealed that these three areas were large multiporous olfactory sensilla (MOS) resembling the pore-plate sensilla (figure 5 A–B). The porous cuticle was considerably thinner than the cuticle of the antennal wall and was crossed by pore canals connecting the external pores with the lumen of the sensillum. Below the porous cuticle, a striking number of dendritic projections completely filled the sensillar lumen (figure 5 C–D). At the level of the pore canal openings, pore tubules could be found (figure 5 F). The MOS were innervated by an undefined number of sensory neurons, typically grouped in bundles of 4 (figure 5 E).

### Neuroarchitecture of the L3 Antennal Lobes

**Figure 6:**
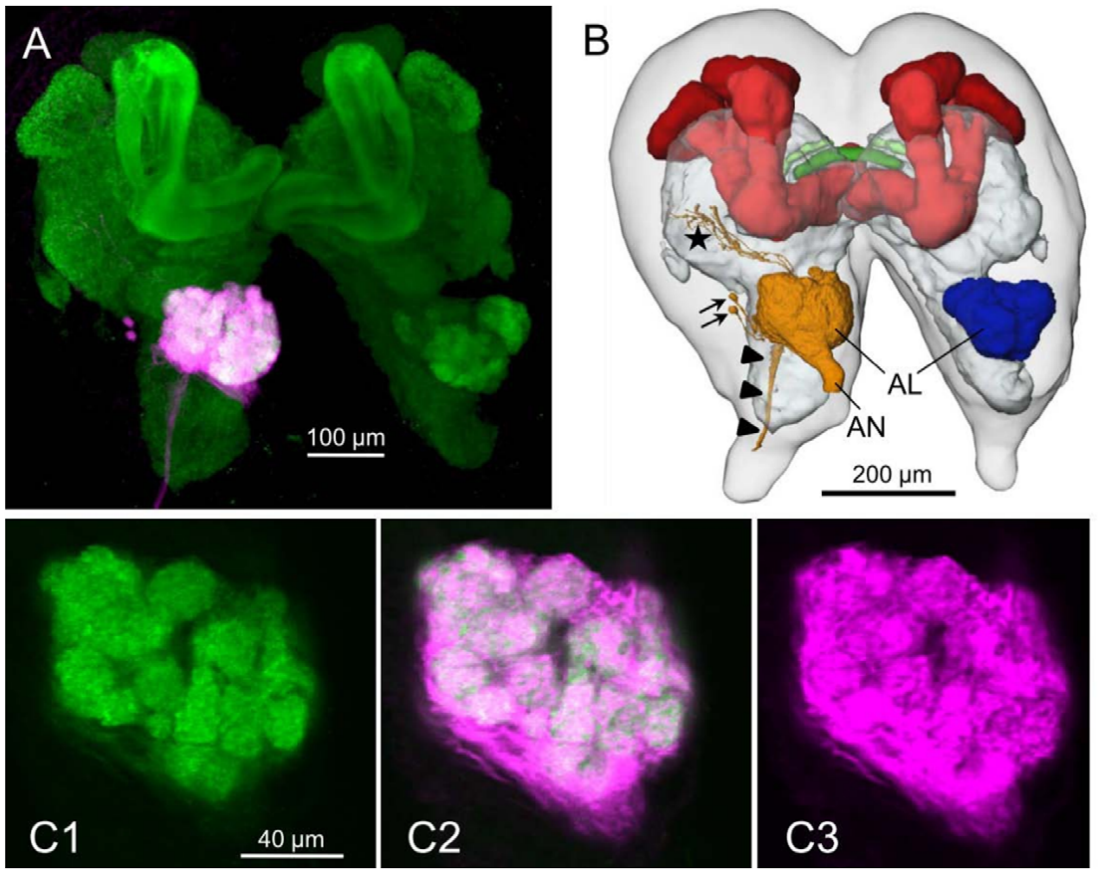
*M. hippocastani* brain including the antennal lobes (AL), frontal views. A) Maximum projection of 229 serial confocal images: Green codes for anti synapsin immunostaining, magenta for a dye (dextran) backfill from the antenna. B) 3D-reconstruction of A showing the brain outline (light gray) and selected brain areas: yellow, reconstructed from the antenna backfill; the other brain areas, including the contralateral AL (blue), the mushroom bodies (red), the central complex (darker green), the protocerebral bridge (lighter green), and remaining neuropil (gray) are reconstructed from the anti synapsin immunostaining which can be used to label neuropil areas in insects (see e.g. [10]). Arrowheads, projection to the subesophageal ganglion; arrows, cell bodies of two antennal motoneurons; star, projections to the lateral protocerebrum; AN, antennal nerve. C) Single confocal images of the image stack of the left antennal lobe in A, clearly showing many spheroidal structures, the so called olfactory glomeruli in the larval beetle brain. AL - labeled by the synapsin antibody (C1) and the backfill staining (C3). C2: Overlay of C1 and C3.

Immunostaining against the ubiquitous synaptic vesicle protein synapsin and antennal backfills revealed a typical insect like glomerular organization of the antennal lobe of third instar *M. hippocastani* with about 70 olfactory glomeruli (figure 6). The backfills showed projections only in the ipsilateral AL but no projections to the contralateral AL as has been described for the majority of OSNs in *Drosophila* (reviewed in [21]). The antennal backfills additionally revealed two cell bodies lateral to the AL, very likely belonging to motoneurons innervating antennal muscles, and projections to the lateral protocerebrum and the subesophageal ganglion (SEG) (figure 6). Antennae are multimodal sensory appendages and house different sensilla with receptor neurons detecting different sensory modalities including mainly olfactory but also contact chemosensory, mechanosensory, temperature and humidity information (e.g. [22], [23]). While OSNs typically project into the olfactory glomeruli of the AL, the mechanosensory axons typically project into a deutocerebral area posterior to the glomerular area called the antennal mechanosensory and motor center (AMMC) or dorsal lobe (reviewed in [23]). The axons of the contact chemoreceptors project into the AMMC but also to the SEG and even further to the thoracic ganglia [24]–[26]. While the projections towards the SEG might thus belong to contact chemoreceptors, the source of the projections to the lateral protocerebrum remains unclear but is unlikely to be OSNs. OSNs in insects seem to exclusively project to the AL (for review see [27]).

### Behavioral Tests

The attraction of *M. hippocastani* larvae to dilutions of pure reference compounds released by oak roots was tested in dual choice tests. The reference compounds were diluted in silicon oil to the concentration producing headspace concentrations as found in the root volatile measurements (Table 1), and tested against the pure silicon oil. In all experiments, we observed no differences in the behavior of the two larval instars (p≥0.05, n.s., chi^2^ test, α = 0.05). Therefore the data were pooled.

**Figure 7:**
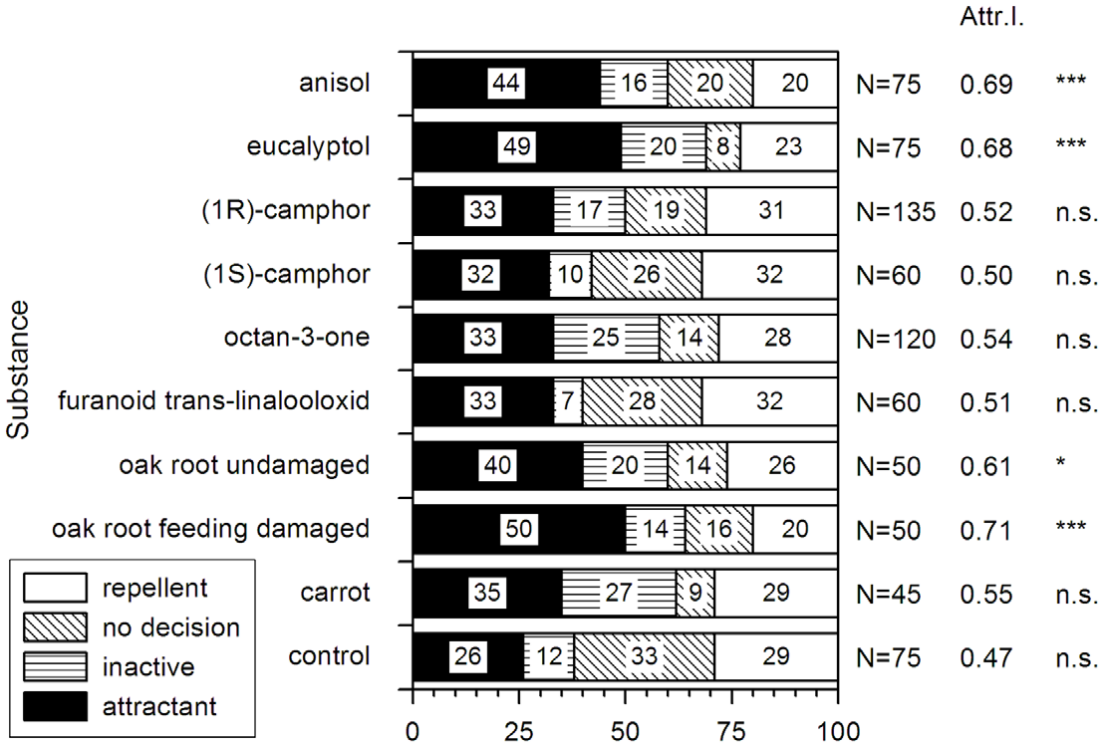
Behavioral data of *M. hippocastani* in dual choice tests in soil. Numbers in the bars show the percentage of larvae orienting towards the odor source (black), of active larvae showing no decision (vertically hatched), of inactive larvae (diagonally hatched), and of larvae orienting away from the odor source (white). Numbers next to the bars indicate the total number of individuals in the different experiments. The Attractivity Index (Attr. I.) was calculated by relating the number of larvae attracted to the compound to the total number of larvae showing a decision. Statistical significance is indicated by *** (p<0.001), * (p<0.05) and n.s. (p>0.05, not significant, chi^2^ test, α = 0.05).

In the control run no preference for one of the two directions could be observed (p≥0.05, n.s., chi^2^ test, α = 0.05). Carrot slices, furanoid trans-linalooloxide, octan-3-one, (1S)-camphor and (1R)-camphor (in each case p>0.05, chi^2^ test) showed no clear attractant or repellent effect. Experiments with anisol and eucalyptol (figure 7) showed similar attractant effects as feeding-damaged oak roots (each with p<0,001, chi^2^ test). Undamaged oak roots apparently exerted a weak yet significant attracting effect on the larvae.

## Discussion

Volatile emissions of the above ground parts of *Quercus* have been investigated by several groups (e.g. [28], [29] and references therein). The adults of *M. hippocastani* were attracted by green leaf volatiles (GLV) and 1,4-benzoquinone as the species-specific sex-pheromone [30]. Experiments documented in [31] showed that orientation behavior of *M. melolontha* larvae was guided by CO_2_ gradients (also shown in [9]), but it changed if plant roots or root exudates were present. Carbon dioxide ceased to be attractive when rhizosphere compounds were added. Thus, the authors hypothesized an interfering or “masking” effect of plant roots or root exudates to the attraction by CO_2_. As a consequence, the authors concluded that additional chemical stimuli beyond CO_2_ might be involved in the localization of host plant roots by *M. melolontha* larvae.

The blend of compounds released by damaged plants might depend on the type of damage [32], [33] and on the animal causing the damage by way of its specific feeding habits [34], [35]. To the best of our knowledge, this is the first time that different kinds of damage have been compared with regard to their effect on the composition of root volatile emissions and their detection by root-feeding insects.

In this study, the trees were manipulated as little as possible in order to maintain the natural character. In the case of the washed root experiments only, the roots were subjected to three alternative and careful treatments prior to sampling the volatiles. However, merely removing the soil particles and washing the roots with tap water might influence the volatile pattern. Moreover, the physiological status of the tree and the organisms living on the tree (on the roots and in the surrounding soil, as well as on the parts above ground) might have some additional impact on the volatile composition (e.g. [36], [37]). However, our results clearly indicate that root derived VOC depend on the kind of damage to a similar extent as shoot-derived VOC [32], [34], [38]. Quantitatively, the emission rates of anisol and eucalyptol from feeding-damaged oak roots were in a similar range as the emission rates of (E)-ß-caryophyllene from maize plants infested by *Diabrotica virgifera* [2].

Our study confirmed that it might be difficult to simulate below-ground herbivory by mechanically damaging the roots [33]. Only one compound was released consistently after both mechanical and herbivore damage.

GC-MS/EAD-experiments with antennae of *M. hippocastani* larvae were performed to identify volatiles released by oak roots which were perceived by the insect. The responses of the antennae did not differ significantly between different larval instars (L2, L3). The compounds, selected on the basis of those GC-MS/EAD experiments, yielded dose-dependent responses with detection limits down to the low ppbv-range.

Thus, white grubs were able to perceive volatile compounds like eucalyptol and anisol specifically released by oak roots damaged by conspecifics at levels of 5 ppbv in soil air. This considerable degree of sensitivity was achieved by six areas containing large pore-plate sensilla on the two distal antennomeres of the *M. hippocastani* larvae. Comparable sensilla structures were shown to detect volatile compounds in the sister species *Melolontha melolontha* [39].

The typical adult olfactory pathway in insects consists of olfactory sensilla mainly on the antennae which house olfactory sensory neurons (OSNs). OSN axons project via the antennal nerve into the antennal lobes (AL), the first central unit for olfactory information processing in the insect brain. From the AL, odor information is then conveyed to higher integration centers including the mushroom bodies and the lateral protocerebrum (reviewed in [27]).

The neuroarchitecture of the olfactory pathway in 3^rd^ instar larvae of *M. hippocastani* clearly resembled the anatomy of a typical adult insect olfactory system (reviewed in [27]). This also compares to findings in the last larval instar of another beetle, *Tribolium castaneum* (Schachtner, unpublished results). The antennae in the 3^rd^ instar larvae of *M. hippocastani* bear three large pore plate sensillae at the apical segment and one on the subapical antennomere which accommodate a large number of OSNs. The sensory neurons are grouped into bundles of 4 sensory neurons, each one ensheathed by its own dendrite sheath. This organization of the olfactory sensilla was reported also in other groups (Homoptera, [40]), for which an origin has been hypothesized as being merged, primarily isolated *sensilla basiconica* [41]. The high number of sensory neurons, associated with the large antennal surface occupied by the pore plates suggests that a key role is played by the olfaction in these below-ground larvae. The axons of the OSNs innervate via the antenna the larval AL. Anti-synapsin immunostaining and antennal nerve backfills revealed in the 3^rd^ instar of *M. hippocastani* ALs containing about 70 glomeruli. The glomeruli are regarded as the functional subunits of odor discrimination [42]. The number of glomeruli compares to glomeruli numbers found in moths or adult beetles (for a review see [27], *Tribolium castaneum*: [43], *Holotrichia diomphalia*: [44]) and thus clearly indicates a highly developed odor processing ability of the cockchafer larvae.

The behavioral experiments with selected volatiles showed that root volatiles such as anisol and eucalyptol elicited significantly attractive responses by *M. hippocastani* larvae moving in soil. The fact that we could detect these compounds by passive sampling of soil air both in 10 cm distance of feeding-damaged oak roots and within the behavioral test arena in comparable concentrations suggests that anisol and eucalyptol are able to diffuse in soil and may serve as a clue for larval orientation.

*M. hippocastani* larvae are frequently found to be clustered in forest stands [45], but this effect has not been attributed to root-borne volatiles. Because of the observed strong attractive effects of root volatiles induced by feeding of conspecifics on host plants, it seems possible that anisol and eucalyptol are part of the signals that can be used by *M. hippocastani* larvae to aggregate.

Several reasons could account for the aggregation of larvae on roots already damaged by conspecifics. Firstly, feeding on roots where conspecifics are feeding might result in stronger growth of the larvae [2]. Secondly, the pronounced aggregation behavior observed during autumn/winter season might help the larvae to find a suitable host tree for overwintering. Such kairomonal activity of root volatiles to below-ground herbivores was already suggested by [1] and [2]. Above ground living insects are known to be affected in their behavior by the presence of plant background odor [46], [47], [48]. However, in contrast to Robert et al. (2012) suggesting for *Diabrotica virgifera virgifera* that “…semiochemicals are only active in the presence of plant background odor”, *M. hippocastani* larvae did respond behaviorally to single compounds. However, this contradiction might be resolved considering that our choice-tests were performed in original soil matrix. This soil matrix might have supplied the necessary amount of background volatiles as habitat odor for the larvae. Thus, it might be advisable to perform behavioral tests of soil dwelling insect instars in original soil substrate in order to overcome problems with unnatural behavior caused by missing habitat odors. Other compounds showing electrophysiological but no behavioral activity in our experiments might modify behavior if present in certain mixture ratios. Thus, further behavioral experiments with odor mixtures will have to be performed in order to gain a more complete understanding of the role of these compounds in directing movements of the cockchafer larvae below ground. However, it is remarkable that natural concentrations of single compounds such as anisol and eucalyptol were able to attract larvae in almost equal numbers like original feeding-damaged roots. Thus, possible additional effects of CO_2_ and other compounds on the choice behavior of the larvae seem not to be necessary to elicit significant attraction. Two grams of carrots (*Daucus carota ssp. sativus*) cut into pieces had no significantly attractive effect on the larvae, although CO_2_ was released from the carrot pieces (data not shown) together with carrot-root specific compounds [49]. In contrast, one gram of feeding-damaged oak roots had a strongly attractive effect on the larvae, CO_2_ being released from the roots to a comparable extent. These few examples of behaviorally active root-borne odor compounds might supply a first hint to understanding the complex interactions between different soil dwelling species that can be elicited by VOCs, opening up a new perspective on the interactions in the below ground world and, from an applied perspective, on the control of soil dwelling insect pests.
